# Supplementary figures and images for: Circular RNA profiling revealed an evolutionarily conserved circACACA promotes liver lipid metabolism, oxidative stress, and autophagy disorder in a ceRNA manner
Source: PLoS Genet. 2025 Dec 18;21(12):e1011729. doi: 10.1371/journal.pgen.1011729 (PMC12753083; doi:10.1371/journal.pgen.1011729)

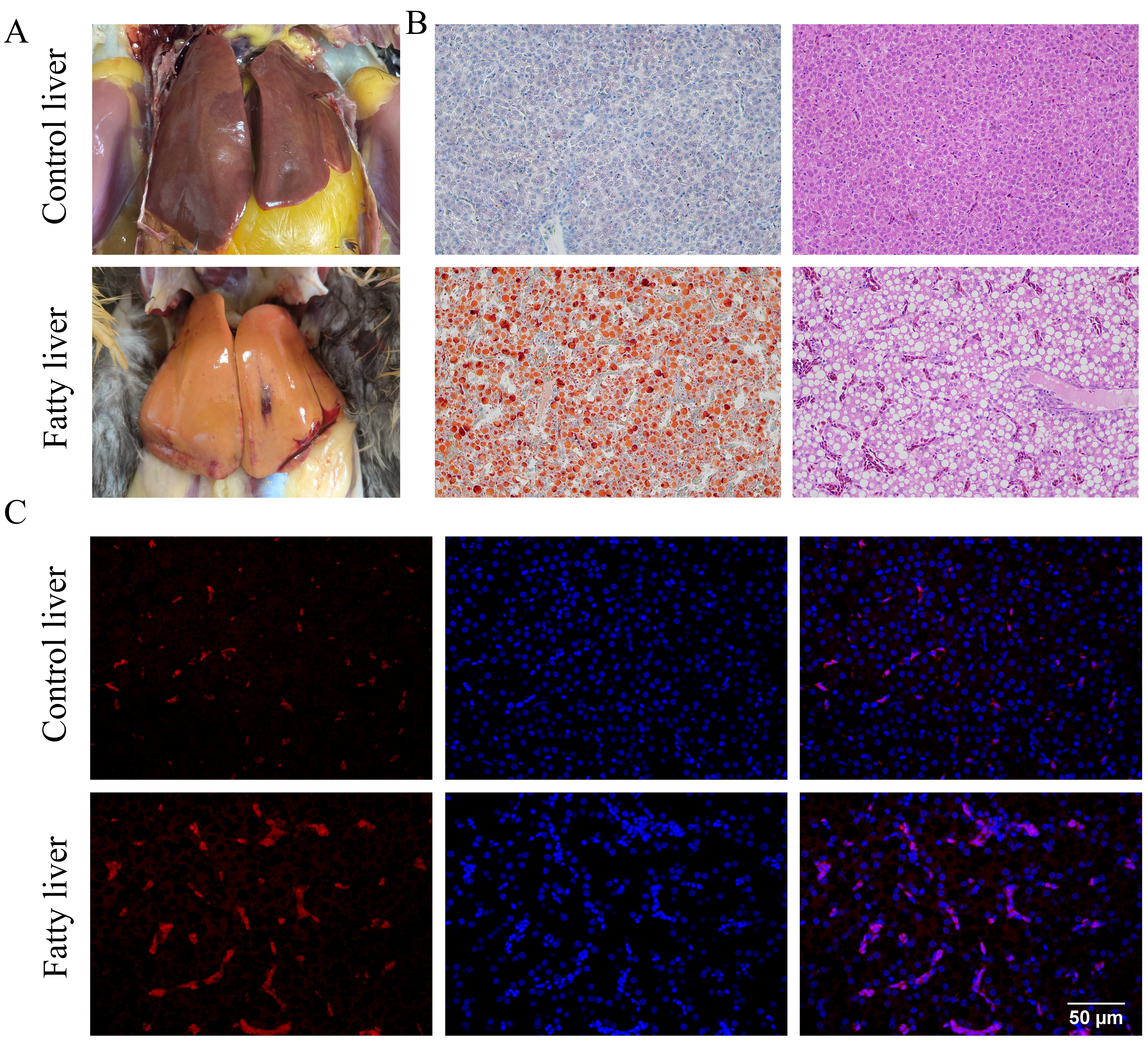

Supplement: S1 Fig — (A) Appearance of normal and fatty liver; (B) Oil Red O staining and HE of liver from both groups of chicken; (C) Immunofluorescence staining images of LC3 in FL and CL. Control liver: CL, Fatty liver: FL. (TIF) [file pgen.1011729.s013.TIF]

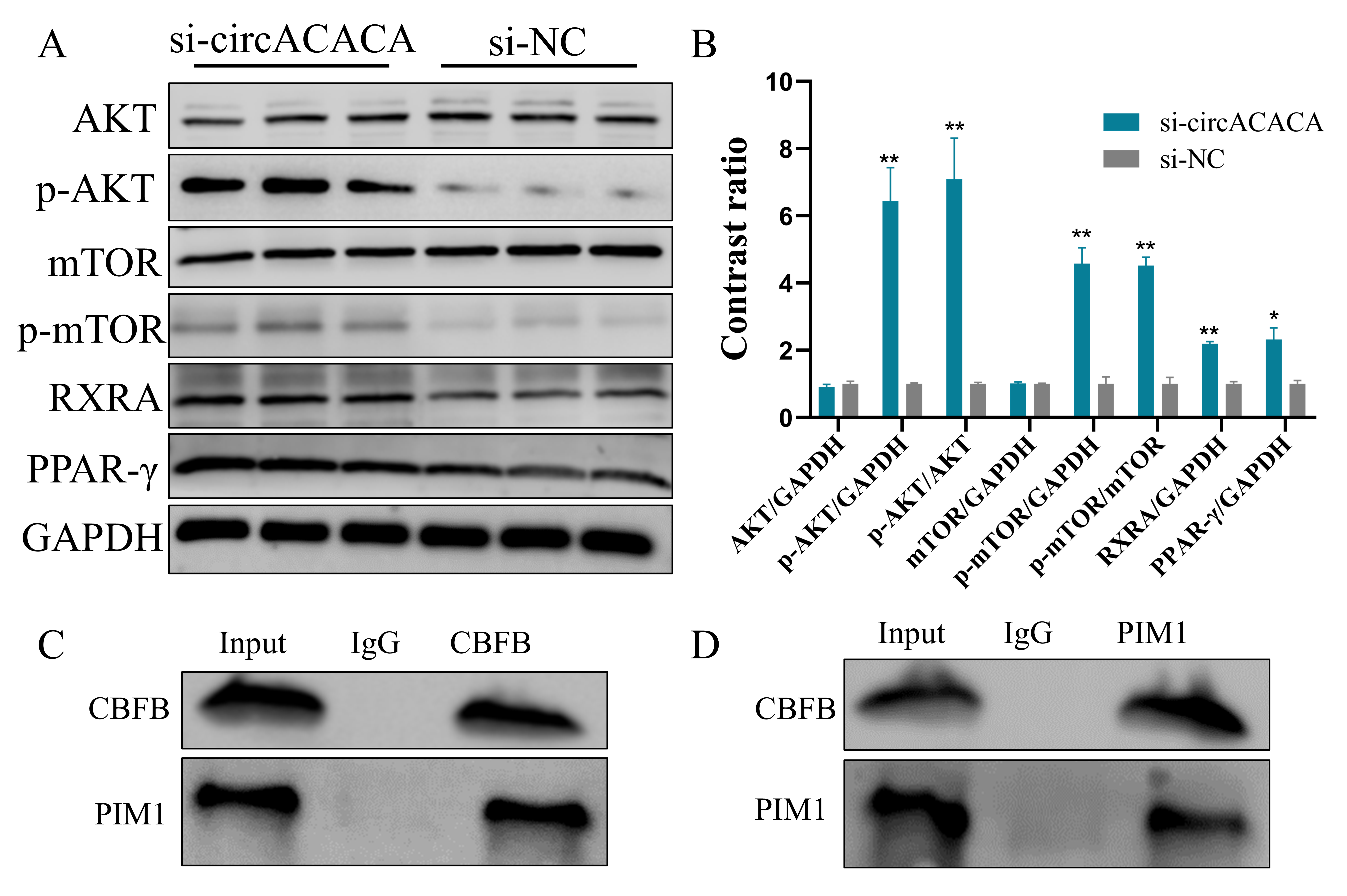

Supplement: S10 Fig — (A) Western blot assessment of the levels of key protein in the AKT/mTOR (AKT, p-AKT, mTOR, p-mTOR) and the PPAR-γ (PPAR-γ and RXRA) signaling pathway after knockdown and overexpression of circACACA. (B) Statistics of the gray value of key proteins in the AKT/mTOR and the PPAR-γ signaling pathway following blocking and increasing circACACA. (C) Co-IP validation of the protein level of CBFB and PIM1 after overexpression of CBFB. (D) Co-IP validation of the protein level of CBFB and PIM1 after overexpression of PIM1. The data is represented as mean ± SEM. * P < 0.05; ** P < 0.01. (TIF) [file pgen.1011729.s022.tif]
